# Splenectomy has opposite effects on the growth of primary compared with metastatic tumors in a murine colon cancer model

**DOI:** 10.1038/s41598-024-54768-5

**Published:** 2024-02-24

**Authors:** Yuki Kaneko, Hideyo Miyato, Mineyuki Tojo, Yurie Futoh, Kazuya Takahashi, Yuki Kimura, Akira Saito, Hideyuki Ohzawa, Hironori Yamaguchi, Naohiro Sata, Joji Kitayama, Yoshinori Hosoya

**Affiliations:** 1 Department of Surgery, Jichi Medical University, Yakushiji 3311-1, Shimotsuke, Tochigi 329-0498 Japan; 2 Department of Clinical Oncology, Jichi Medical University Hospital, Shimotsuke, Japan

**Keywords:** Cancer, Immunology

## Abstract

The spleen is a key source of circulating and tumor-infiltrating immune cells. However, the effect of splenectomy on tumor growth remains unclear. At 3 weeks after splenectomy, we subcutaneously injected LuM1 cells into BALB/c mice and evaluated the growth of primary tumors and lung metastases at 4 weeks after tumor inoculation. In addition, we examined the phenotypes of immune cells in peripheral blood by using flow cytometry and in tumor tissue by using multiplex immunohistochemistry. The growth of primary tumors was reduced in splenectomized mice compared with the sham-operated group. Conversely, splenectomized mice had more lung metastases. Splenectomized mice had fewer CD11b^+^cells, especially monocytic MDSCs (CD11b^+^Gr-1^neg-low^Ly6c^high^), and NK cells (CD49b^+^CD335^+^). The proportion of NK cells was inversely correlated with the number of lung metastases. In splenectomized mice, the density of CD3^+^ and granzyme B^+^ CD8^+^ T cells was increased, with fewer M2-type macrophages in primary tumors, but NK cells were decreased markedly in lung. Splenectomy concurrently enhances T cell-mediated acquired immunity by reducing the number of monocytic MDSCs and suppresses innate immunity by decreasing the number of NK cells. Splenectomy has opposite effects on primary and metastatic lesions through differential regulation on these two immune systems.

## Introduction

The spleen is the largest lymphoid organ, and its numerous immune cells play complex roles in immune surveillance and immune defense against infection and cancer^1^. Splenectomy is often performed during upper abdominal surgery for trauma or cancer treatment. Because of recent advances in understanding tumor immunology, the exact contribution of splenectomy to patient outcome is attracting interest. Epidemiologic studies indicate that splenectomy for trauma has little, if any, effect on the subsequent occurrence of cancers^2,3^. However, some clinical studies suggest that splenectomy negatively affects the survival of patients with gastric^4,5^, colonic^6^, or pancreatic^7^ cancers. Yet, these findings might reflect confounding bias associated with the clinical indication for splenectomy, including locally advanced tumors and increased surgical stress. Therefore, whether splenectomy increases the risk of cancer growth or recurrence in human patients is unclear.

Murine studies of the effects of splenectomy on tumor development and progression have yielded highly conflicting results. In some studies, splenectomy slightly suppressed the growth of subcutaneously^8,9^ or orthotopically^10^ inoculated tumors. Other studies have reported nonsignificant differences^11–14^ or even tumor- growth-promoting effects^15,16^. In metastatic models, splenectomy has been reported to either enhance^8,11,14–19^ or inhibit^9,13,20–22^ the formation of lung or liver metastases. These discrepancies have been attributed to the diversity of experimental models used, including differencies in tumor cell type, and mouse strain, and in the methods for evaluating anti-tumor effects. In particular, the effects of splenectomy on tumor growth are highly dependent on the timing of splenectomy relative to tumor inoculation^9,20,23,24^. In the same experimental series, splenectomy has differently affected the growth of primary tumors and metastases^8,11,13,14,17^.

The spleen is a key reservoir and source of circulating and tumor-infiltrating immune cells^25,26^. In particular, the frequencies of immunosuppressive myeloid-derived suppressor cells (MDSCs), which disrupt both adaptive and innate immune cell responses^27,28^, are increased in the spleens of tumor-bearing mice^13,22^. The spleen is an important extramedullary organ that continuously supplies neutrophils and monocytes to tumor sites and thus promotes tumor growth^29^. Together, these findings indicate that splenectomy changes the dynamics of myeloid cells and thus has differing effects on primary and metastatic lesions.

Most of these previous studies used metastatic mouse models created through intravenous or intrasplenic injection of cancer cells. As such, these models do not adequately recapitulate effects on tumor metastasis, given that metastasis is a highly complex process in which cancer cells detached from the primary tumor must escape immune surveillance pathways^30^. Some studies indicate that the primary tumor controls the development and growth of metastases by modifying of immunologic status^31,32^. Here, we subcutaneously inoculated mice with LuM1 cells, a highly metastatic subclone of a murine colon adenocarcinoma^33^, and evaluated the effects of splenectomy on the growth of primary tumors and on metastatic lesions in the lung.

## Results

### Splenectomy suppressed the growth of primary (subcutaneous) tumors

**Figure 1:**
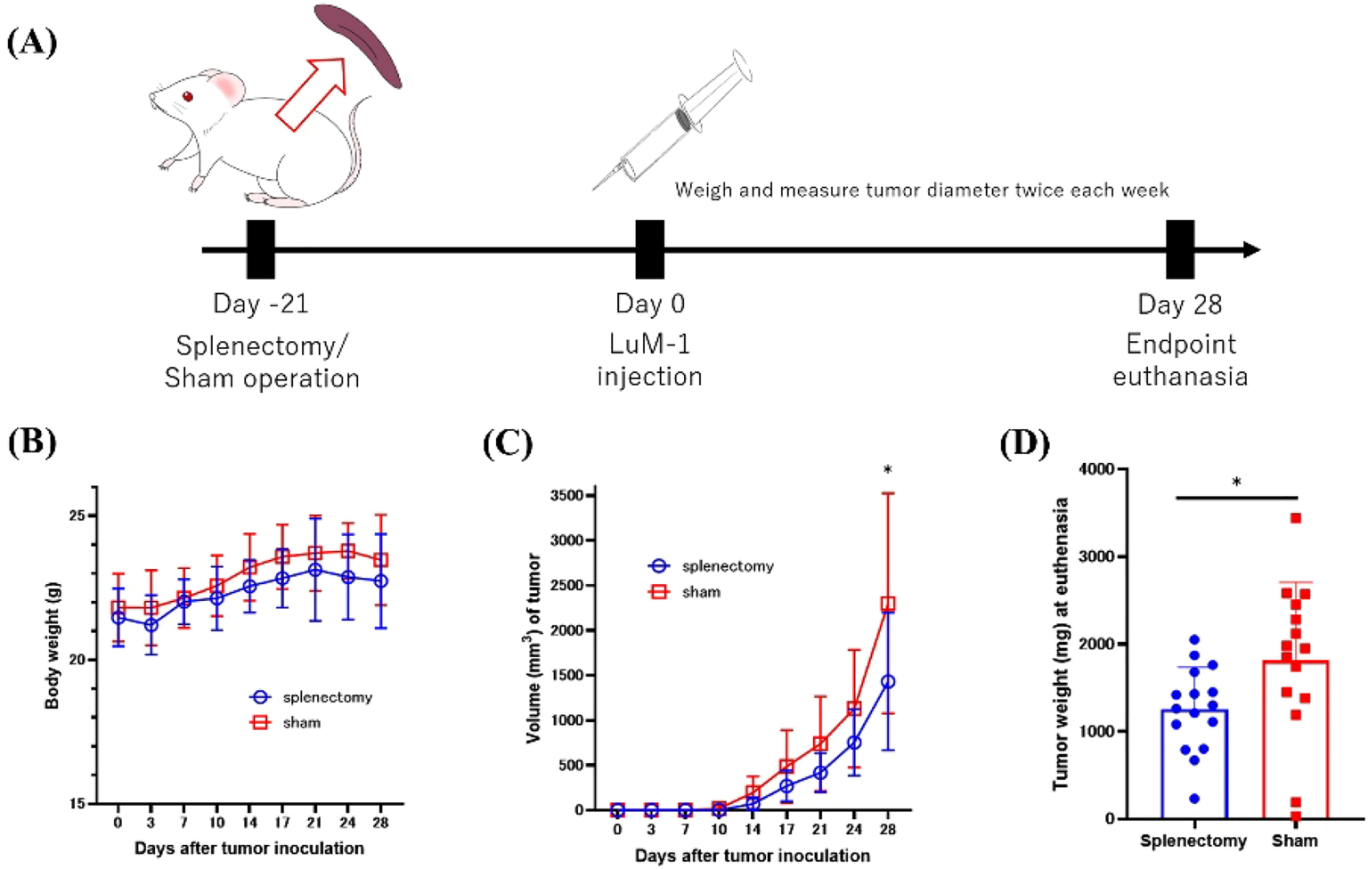
Experimental scheme and primary tumor growth. (**A**) BALB/C mice underwent splenectomy or a sham operation prior to subcutaneous injection of LuM1 colon adenocarcinoma cells (1 × 10^6^ per mouse) in the right flank. (**B**) Body weight and (**C**) tumor volume were measured twice each week after tumor inoculation. On day 28, mice were euthanized, and (**D**) primary tumors were weighed. The combined results of 2 experiments (n = 7 or 8 mice in each group) are shown. *P < 0.05 (Mann–Whitney *U* test).

Tumors were detected in all mice at about 10 days after subcutaneous injection of LuM1 colon adenocarcinoma cells. Weight trends did not show significant differences between each group (Fig. 1B). Splenectomy prior to tumor inoculation slightly but significantly suppressed the growth of primary (subcutaneous) tumors (Fig. 1C, D). In the splenectomy group, the median tumor volume after euthanasia at day 28 (1400 mm^3^; range, 174–3063 mm^3^, n = 16 per group) was significantly less than that of the sham control group (2604 mm^3^, 44–3934 mm^3^, n = 15, *P* < 0.05). Tumor weight after euthanasia was reduced in the splenectomy group (1280 mg, 230–2050 mg; controls: 1950 mg, 30–3440 mg, *P* < 0.05) also.

### Splenectomy exacerbated spontaneous lung metastases

**Figure 2:**
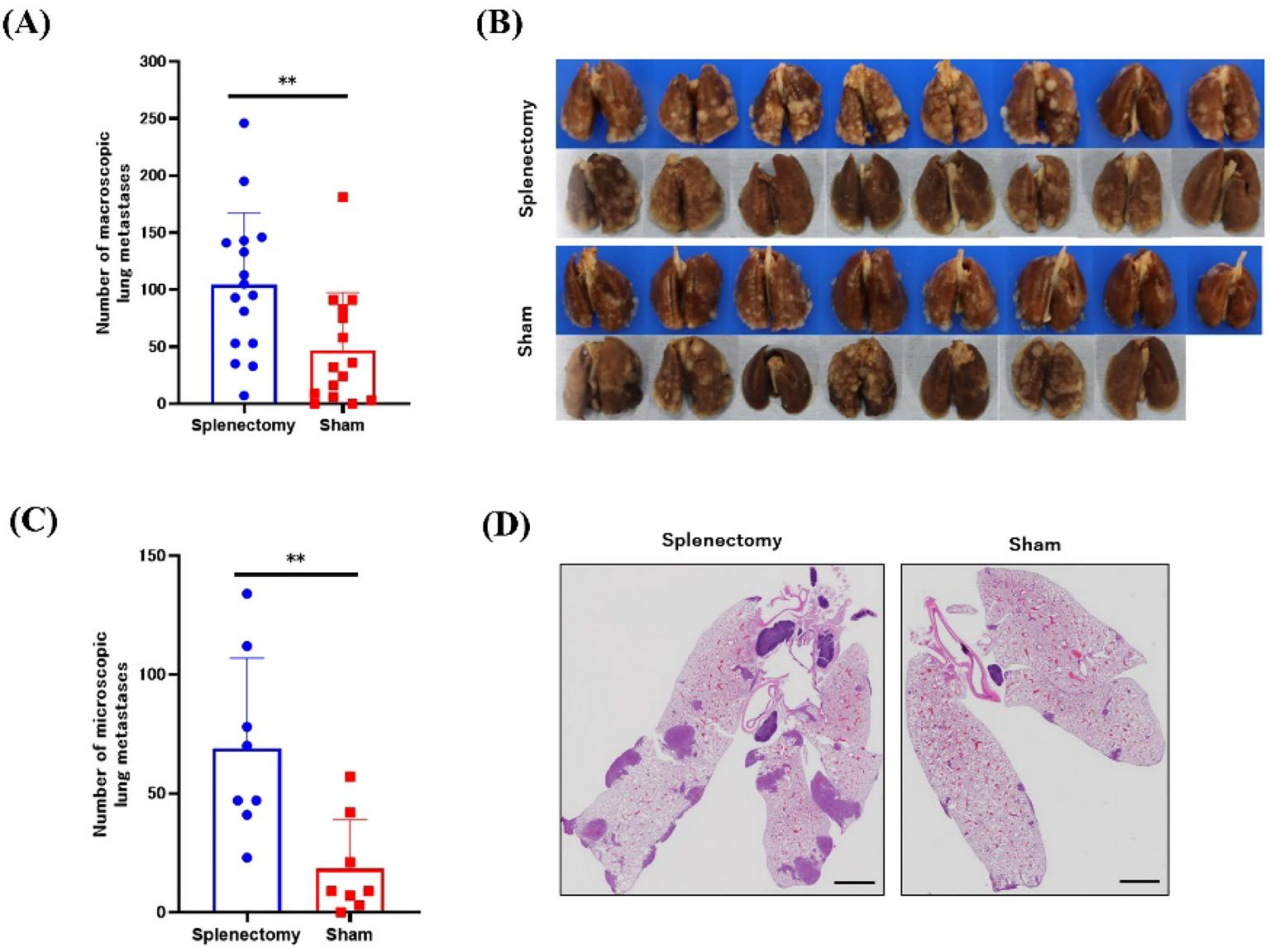
Growth of macroscopic and microscopic metastases in lung. (**A, B**) On day 28, lung metastases were evaluated by counting the number of macroscopic nodules visible at the lung surface. The combined results of 2 experiments (n = 7 or 8 mice in each group) are shown. (**C, D**) Representative coronal tissue sections from the central part of formalin-fixed lung specimens were stained with hematoxylin and eosin, and the total number of lung metastases, including microscopic lesions, was counted. **P < 0.01 (Mann–Whitney *U* test).

By day 28, multiple metastases had developed in the lungs of both groups. However, the median number of macroscopic metastases visible at the lung surface was significantly higher in splenectomized mice (100; range, 7–246) than in control mice (32, 0–181, *P* < 0.01) (Fig. 2A, B). We then used microscopy to evaluate the metastatic lesions including microscopic tumors in a representative coronal tissue section from each mouse. Again, the median number of lung metastases was significantly higher in the splenectomy group (58.5, 23–134) than in controls (9, 0–42, *P* < 0.01) (Fig. 2C, D).

### Splenectomy decreased the frequency of natural killer (NK) cells in the peripheral blood of LuM1 tumor-bearing mice

**Figure 3:**
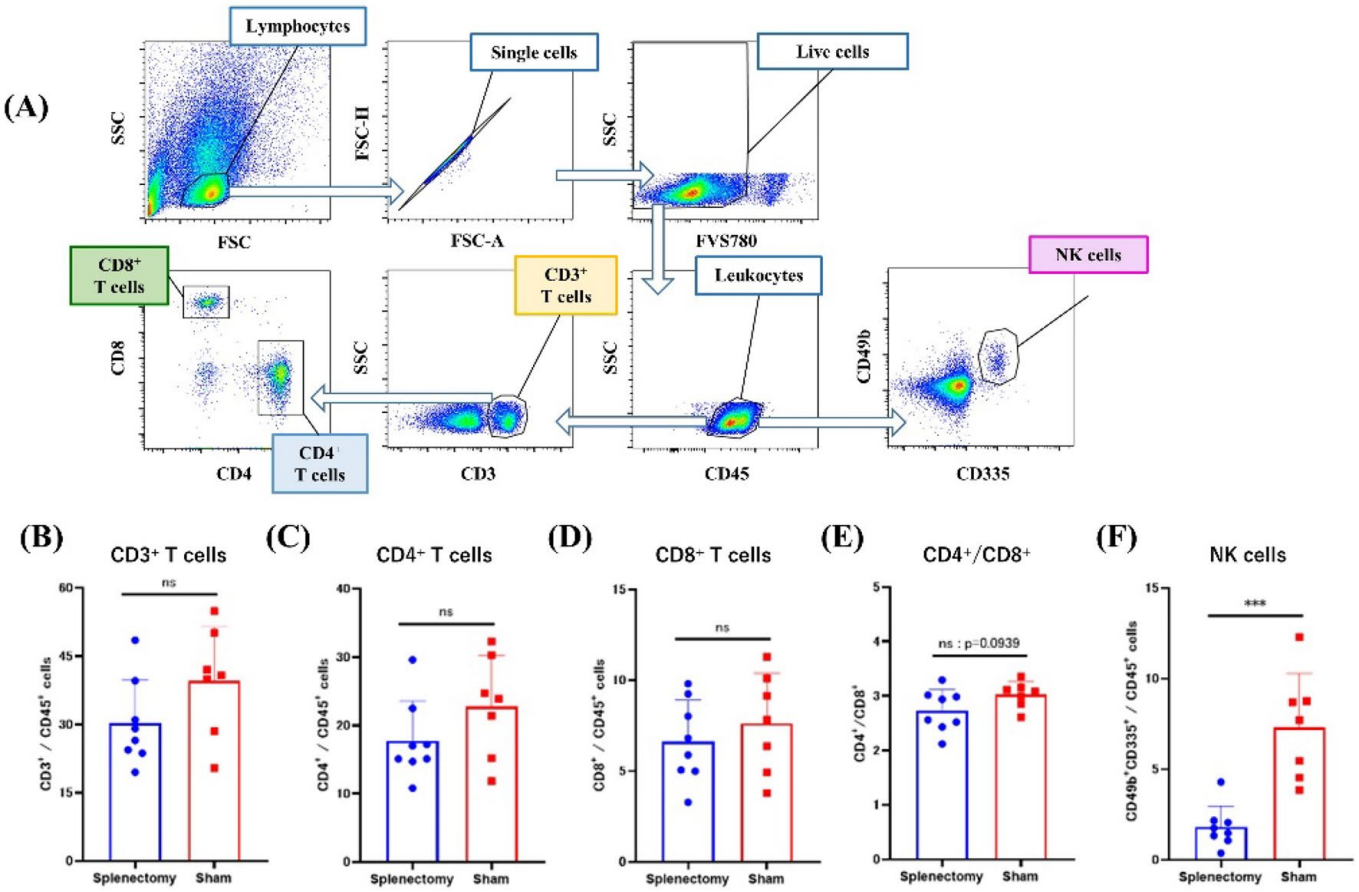
Frequencies of lymphoid cell subsets in blood. (**A**) Gating strategy (FlowJo software) for lymphoid cells in peripheral blood. Among total cells, lymphocytes were distinguished according to the forward scatter/side scatter profile. Circulating immune cells harvested at day 28 were immunostained with monoclonal antibodies to CD3, CD4, CD8, CD45, CD49b, and CD335, and the frequencies of (**B**) CD3^+^ total T cells, (**C**) CD3^+^CD4^+^ T cells, (**D**) CD3^+^CD8^+^ T cells, and (**F**) CD49b^+^CD335^+^ NK cells in CD45^+^ leukocyte population and the (**E**) CD4^+^/CD8^+^ T cell ratio were determined through flow cytometry. ***P < 0.001 (Mann–Whitney *U* test).

Using flow cytometry, we calculated the ratios of T cells and NK cells in CD45(+) cells from peripheral blood at day 28 by gating on lymphocytes (Fig. 3A). Neither the frequencies of CD3^+^, CD4^+^, and CD8^+^ T cells nor CD4^+^/CD8^+^ ratios differed between splenectomized and control mice (Fig. 3B–E). However, the median frequency of NK cells, characterized as CD49b^+^CD335^+^ cells, was markedly lower in the splenectomy group (1.61%, 0.37–4.30%) than in the sham control group (7.71%, 3.85–12.30%, *P* < 0.001) (Fig. 3F).

### Splenectomy decreased the frequencies of myeloid cells, especially CCR2^+^ monocytic MDSCs (M-MDSCs), in the peripheral blood of LuM1-bearing mice

**Figure 4:**
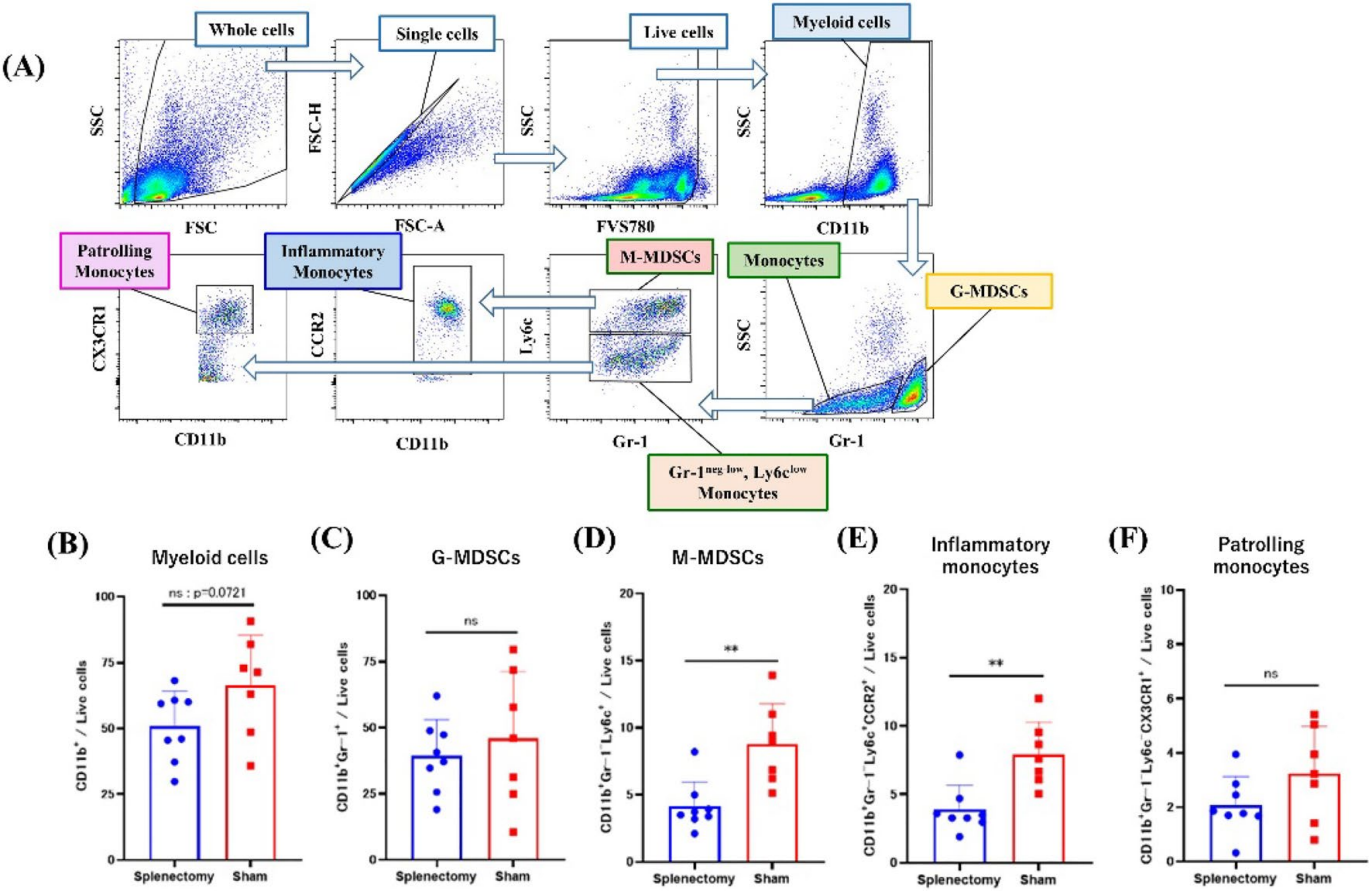
Frequencies of myeloid cell subsets in blood. (**A**) Gating strategy (FlowJo software) for myeloid cells in peripheral blood. Dead cells were excluded by using FVS780. Peripheral immune cells harvested at day 28 were immunostained with monoclonal antibodies to CD11b, Gr-1, Ly6c, CCR2, and CX3CR1, and the frequencies of (**B**) CD11b^+^ myeloid cells, (**C**) CD11b^+^Gr-1^+^ granulocytic MDSCs, (**D**) CD11b^+^Gr-1^–^Ly6C^+^ monocytic MDSCs, (**E**) CD11b^+^Gr-1^–^Ly6C^+^CCR2^+^ inflammatory monocytes, and (**F**) CD11b^+^Gr-1^–^Ly6C^–^CX3CR1^+^ patrolling monocytes in the live cell population were determined by using flow cytometry. **P < 0.01 (Mann–Whitney *U* test).

We then assessed the proportions of various myeloid cell subsets in live cells from peripheral blood. (Fig. 4A). Overall, the ratios of CD11b^+^ myeloid cells tended to be decreased in the splenectomy group (*P* = 0.0721) (Fig. 4B). In particular, the subpopulation of CD11b^+^Gr-1^–^Ly6C^+^ M-MDSCs was significantly smaller in the splenectomy group (3.61%, 2.11–8.20%) than in control mice (8.89%, 5.12–13.9%) (Fig. 4D), but the ratio of CD11b^+^Gr-1^+^ G-MDSCs did not differ between groups (Fig. 4C).

Recent studies have suggested that monocytes can be classified into inflammatory classical (CCR2^high^ Ly6C^+^) and patrolling nonclassical (CX3CR1^+^ Ly6C^–^) subtypes, with different dynamics and functions^34^. Patrolling monocytes differentiate from classical monocytes through Notch signaling primarily in bone marrow and spleen^35^ and play critical roles in the elimination of circulating tumor cells^36^. We therefore examined the frequencies of inflammatory and patrolling monocytes in peripheral blood. Most of the M-MDSCs showed the inflammatory CCR2^high^ Ly6C^+^ phenotype, and their frequency was significantly lower in splenectomized mice (3.38%, 1.90–7.87%) than in controls (7.59%, 5.04–12.00%, *P* < 0.01) (Fig. 4E). The frequency of CX3CR1^+^Ly6C^–^ patrolling monocytes was also lower in the splenectomy group, but the difference between groups did not reach statistical significance (Fig. 4F). When analyzing the absolute cell numbers, splenectomy and control group exhibited a similar trend in every subpopulation, with a slightly more pronounced difference (Supplementary Fig. 1).

### The number of lung metastases correlated with the frequencies of NK cells and patrolling monocytes in peripheral blood

**Figure 5:**
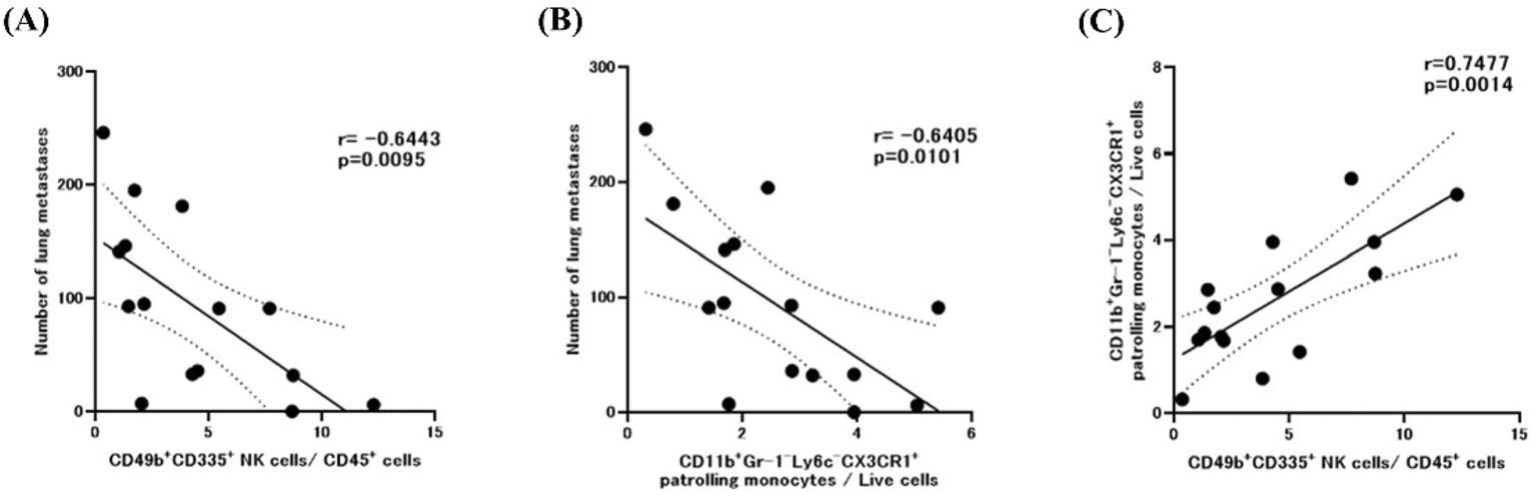
Macroscopic lung metastases and circulating immune cells. Correlation between the number of macroscopic lung metastases and the percentage of (**A**) CD49b^+^CD335^+^ NK cells or (**B**) CD11b^+^Gr-1^–^Ly6c^–^CX3CR1^+^ patrolling monocytes in blood was evaluated by using Pearson’s correlation coefficient. (**C**) The correlation between the percentages of NK cells and patrolling monocytes was evaluated also. Pearson simple linear regression analysis was used to calculate *r* and *P* values.

We then assessed the correlations between the number of lung metastases and various types of circulating immune cells in all mice. The number of lung metastases showed a strong inverse correlation with the frequency of CD49b^+^CD335^+^ NK cells (r = – 0.8646, *P* < 0.01) and CD11b^+^Gr-1^–^Ly6C^–^CX3CR1^+^ patrolling monocytes (r = – 0.6405, *P* < 0.05) (Fig. 5A, B), whereas the frequencies of NK cells and patrolling monocytes showed strong positive correlation (r = 0.7477, *P* < 0.01) (Fig. 5C).

### Splenectomy increased the density of tumor-infiltrating lymphocytes (TILs) but decreased the density of M2-type tumor-associated macrophages (TAMs) in primary tumors

**Figure 6:**
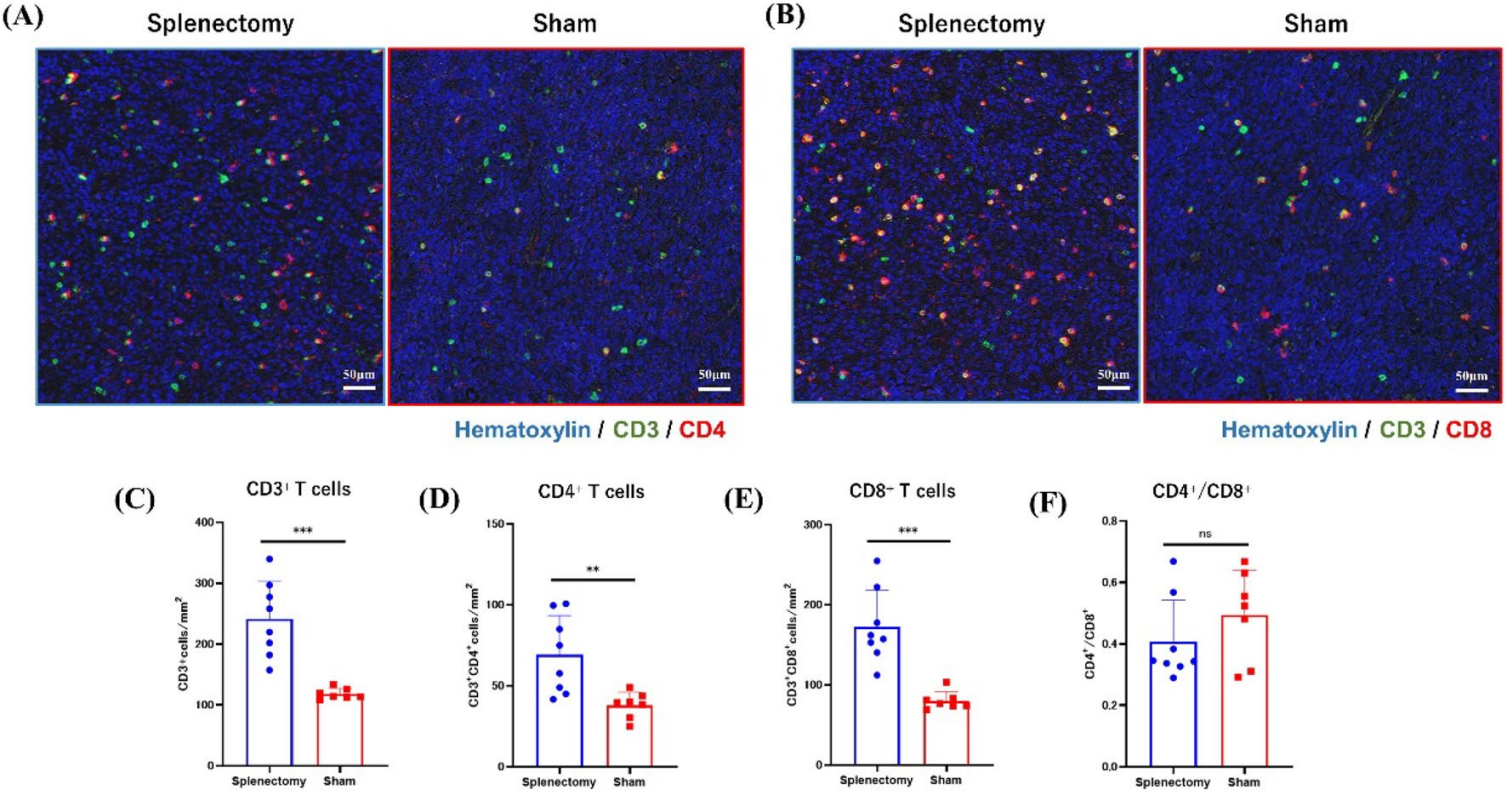
Multiplex immunostaining of tumor-infiltrating lymphocytes (TILs) in primary tumors. (**A**) CD3(green)^+^CD4(red)^+^ and (**B**) CD3(green)^+^CD8(red)^+^ double-positive cells in 3 randomly selected fields (1.0 × 1.0 mm) of primary tumors were counted. (**C–F**) The densities of CD3^+^, CD4^+^, and CD8^+^ TILs and the CD4^+^/CD8^+^ cell ratio were compared between splenectomized and control mice. *P < 0.05; **P < 0.01; ***P < 0.001 (Mann–Whitney *U* test).

We used multiplex immunohistochemistry to examine the effect of splenectomy on TILs and TAMs in subcutaneous primary tumors. Representative images of CD3/CD4 and CD3/CD8 double-positive cells are shown in Supplementary Fig. 2A, B. The density of total CD3^+^ TILs was significantly higher in the splenectomy group (233.8 cells/mm^2^, 157.3–339.6 cells/mm^2^) than in controls (114.0 cells/mm^2^, 108.3–133.6 cells/mm^2^, *P* < 0.001) (Fig. 6A–C), as were the densities of CD4^+^ and CD8^+^ TILs (splenectomy group: CD4^+^ cells: 66.3 cells/mm^2^, 41.7–100.6 cells/mm^2^; control group: 39.6 cells/mm^2^, 25.0–49.0 cells/mm^2^, *P* < 0.01; CD8^+^: splenectomy group, 159.7 cells/mm^2^, 112.3–254.7 cells/mm^2^; control group, 76.7 cells/mm^2^, 69.0–103.3 cells/mm^2^, *P* < 0.001) (Fig. 6D, E). Many CD8^+^ T cells were positive for granzyme B, suggesting the activated effector T cells (Supplementary Fig. 2E), and their density was also higher in splenectomy group (Supplementary Fig. 3). In contrast, CD4^+^/CD8^+^ T cell ratio did not differ between the groups (Fig. 6F). In lung metastatic nodules, the density of CD3^+^ TILs overall tended to be increased (*P* = 0.089) in splenectomized mice, with the CD8^+^ TIL subpopulation significantly increased in the splenectomy group (234.0 cells/mm^2^, 27.0–873.0 cells/mm^2^) compared with the sham group (175.5 cells/mm^2^, 45.0–504.0 cells/mm^2^, *P* < 0.05). CD4^+^/CD8^+^ T cell ratio did not differ between the groups (Supplementary Fig. 4A–F).

**Figure 7:**
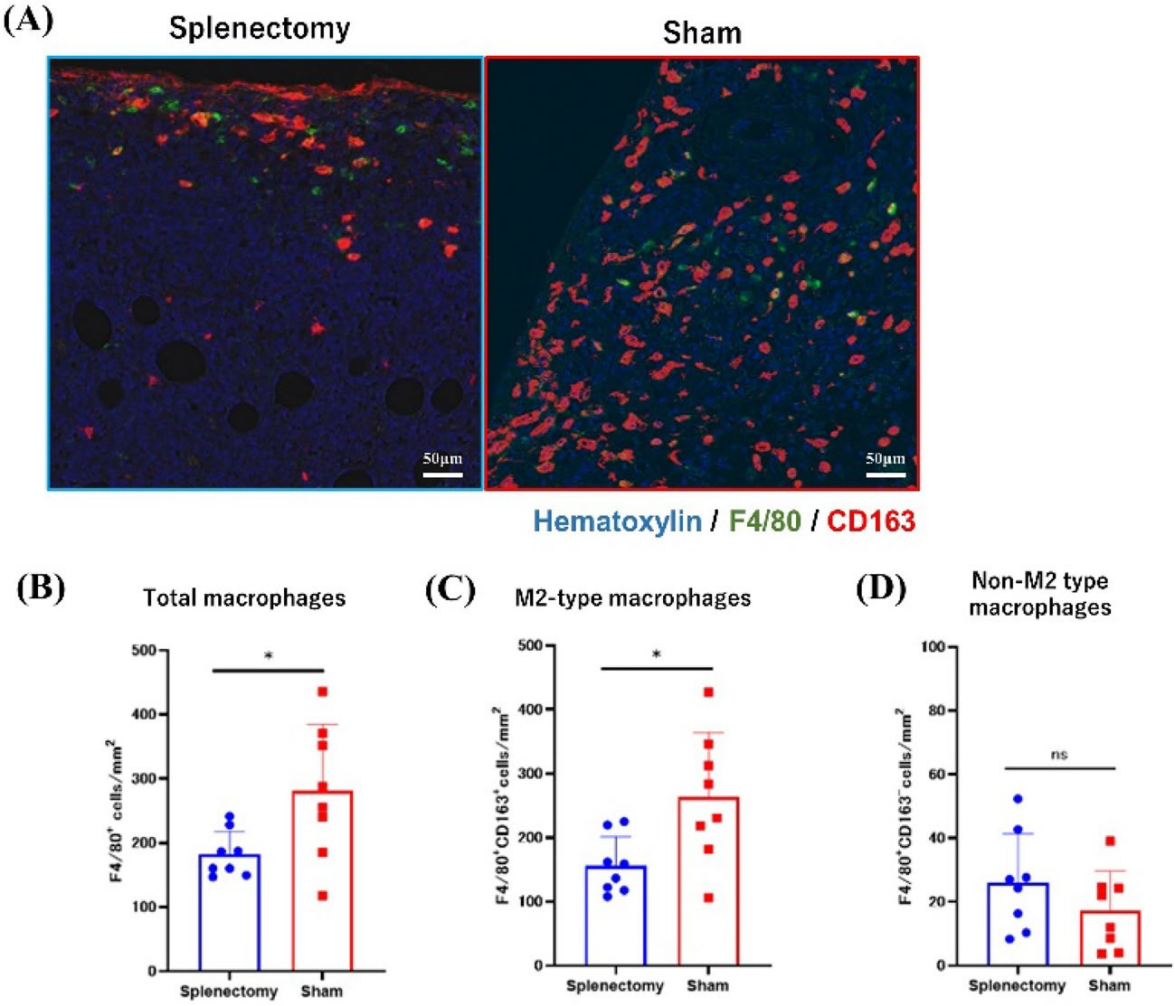
Multiplex immunostaining of tumor-associated macrophages (TAMs) in primary tumors. (**A**) F4/80(green)^+^CD163(red)^+^ double-positive M2-type TAMs and F4/80(green)^+^ TAMs in 3 randomly selected fields (1.0 × 1.0 mm) of primary tumors were counted. The density of TAM (**B**) and each TAM population (**C, D**) was compared between splenectomized and control mice. *P < 0.05 (Mann–Whitney *U* test).

Using the same tissue sections, we quantified TAMs by staining for F4/80 and CD163; M2-type TAMs were defined as F4/80/CD163 double-positive cells (Supplementary Fig. 2C). Whereas the density of M2-type macrophages in primary tumors was significantly lower in the splenectomy group (148.0 cells/mm^2^, 108.0–225.0 cells/mm^2^) than in controls (257.3 cells/mm^2^, 106.0–427.0 cells/mm^2^, *P* = 0.0379), that of F4/80 single-positive TAMs did not differ between the groups (Fig. 7A–D). The lungs were also evaluated for them, however, there was no difference. (Supplementary Fig. 5A–D).

### The density of NK cells was remarkably reduced in the lungs of splenectomized mice

**Figure 8:**
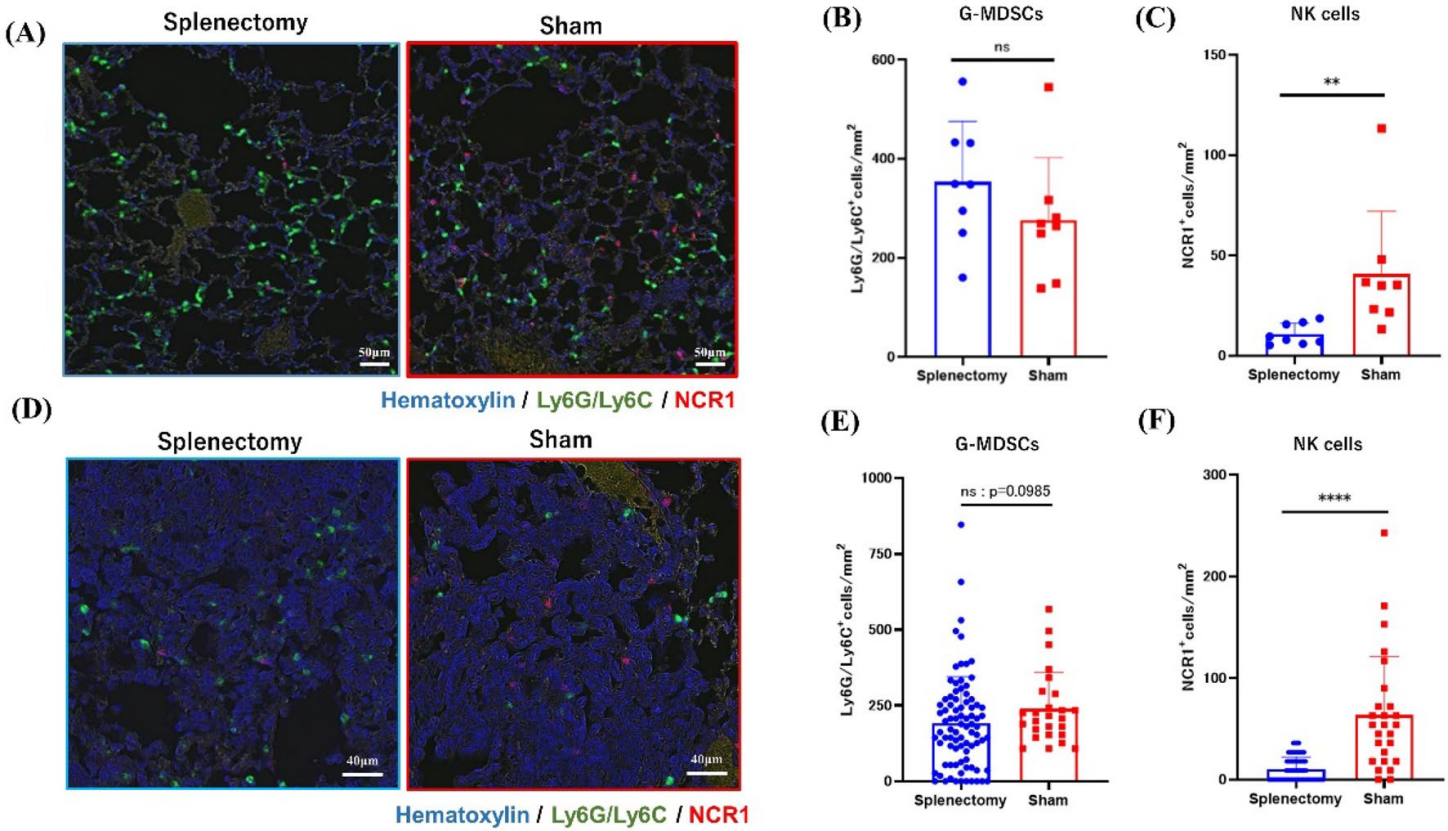
Multiplex immunostaining of granulocytic MDSCs (G-MDSCs) and NK cells in lung. Ly6G/Ly6C(green)^+^ G-MDSCs and NCR1(red)^+^ NK cells in 3 randomly selected fields (**A**) 1.0 × 1.0 mm in lung tissue without metastasis or (**D**) 0.3 × 0.3 mm in metastatic lesions larger than 160,000 µm^2^ (total lesions: 85 in splenectomy group, 26 in control group) were counted. Comparison of the density of (**B**, **C**) G-MDSCs and (**E**, **F**) NK cells between splenectomized and control mice. **P < 0.01; ****P < 0.0001 (Mann–Whitney *U* test).

Finally, we examined the densities of G-MDSCs (identified as Ly6G/Ly6C^+^ cells) and NK cells (NCR1^+^ cells) (Supplementary Fig. 2D) in the lung tissue of our mice. The majority of NCR1^+^ NK cells were positive for granzyme B, suggesting activated NK cells (Supplementary Fig. 2F). In metastasis-free lung tissue, the density of G-MDCSs did not differ between splenectomized and control mice (Fig. 8A, B), but the density of NK cells was markedly lower in splenectomized mice (8.833 cells/mm^2^, 5.3–18.6 cells/mm^2^) than in controls (35.1 cells/mm^2^, 13.3–113.3 cells/mm^2^, *P* = 0.0011) (Fig. 8A, C). In metastatic nodules, the density of G-MDSCs again did not differ between groups, but that of NK cells was markedly lower in splenectomized mice (9.0 cells/mm^2^, 0–36.0 cells/mm^2^) than in controls (54.0 cells/mm^2^, 0–243.0 cells/mm^2^, *P* < 0.0001) (Fig. 8D–F). Similar results were observed for subcutaneous primary tumors (Supplementary Fig. 6A-C).

## Discussion

The influence of splenectomy on tumor development and progression remains inconclusive. In previous murine studies, splenectomy had both suppressive^9,10,20–23,29^ and promoting^8,11,14–16,18,19^ effects on tumor growth, suggesting that the role of the spleen in tumor behavior is largely dependent on cancer cell type and stage. In the present study, we used a spontaneous lung metastasis model and found that splenectomy prior to tumor induction had opposite effects on the growth of primary compared with metastatic lesions: splenectomy slightly suppressed the growth of the subcutaneous primary tumors but exacerbated lung metastases. We observed the same effects of splenectomy on primary tumor and peritoneal metastasis using a gastric cancer cell, YTN16P, and C57BL/6N mouse (Supplementary Fig. 7). Differential effects of splenectomy on primary compared with metastatic sites has previously been reported for other cancer cell types, including melanoma^17^, hepatoma^11^, lung carcinoma^8^, and breast cancers^13,14^. Together, these combined results suggest that splenectomy differently modulates the immune microenvironment in primary tumors compared with the metastatic or premetastatic niche in the target organ.

Consistent with previous studies^9,13,29^, we found that the frequency of peripheral myeloid cells tended to be decreased and the frequency of Ly6C^high^CCR2^+^ M-MDSCs was reduced after splenectomy; these findings confirm the concept that spleen is a major source of circulating and tumor-infiltrating MDSCs. Moreover, we found that M2-type TAMs in primary tumor were reduced and CD4^+^ and CD8^+^ tumor-infiltrating T cells were increased in splenectomized mice, although the frequencies of total T cells and their subsets in peripheral blood did not differ between groups. Considering that a significant proportion of the CD8^+^ TILs exhibit positivity for granzyme B, and their density also exhibited an increase in splenectomized mice, it is reasonable to designate them as the principal activated effector cells responsible for tumor reduction. Previous studies have shown a positive correlation between the CD8^+^ TILs and clinical outcome in many cancers, including colon cancer^37–39^. In addition, our results are supported by the fact that many in vivo experiments have shown increased tumor volume and poor prognosis with the depletion of CD8^+^ T cell using anti-CD8 antibody^40–43^.

Circulating M-MDSCs infiltrate tumors via the CCL2/CCR2 axis and differentiate into M2-type TAMs^44^; this cell population plays a pivotal role in assisting tumor growth by suppressing T cell functions and promoting angiogenesis^28,45^. Blockade of the CCL2/CCR2 axis decreased tumor-infiltrating inflammatory monocytes–macrophages but increased T cell infiltration in a murine pancreatic cancer model^46^. Considering these previous results with our current findings raises the possibility that the decreased number of M-MDSCs after splenectomy may enhance T cell infiltration and activation in the tumor microenvironment, thus inhibiting the growth of primary tumors.

In contrast to the effects on primary tumors, splenectomy markedly accelerated lung metastasis. Similar results have previously been reported in some studies^8,11,14,20^ but not others^9,13,21^. Tumor metastasis is regulated by various factors including cytokines, chemokines as well as bone marrow derived cells. In particular, pro-inflammatory cytokines secreted from tumor cells and host cells are known to play key roles in the formation of pre-metastatic niche^47^. In the current study, we additionally found that the frequency of NK cells, which were positive for CD49b and CD335 (also known as NCR1 and NKp46), was greatly reduced in peripheral blood and in both metastatic lesions and metastasis-free lung tissue in splenectomized mice. The majority of the NK cells in lung were positive for granzyme B, suggesting activated NK cells. Moreover, the number of lung metastases was inversely correlated with the ratio of NK cells. Given that NK cells negatively control metastasis through an NCR1-dependent mechanism^48–50^, the reduced number of NK cells after splenectomy likely is responsible for the metastasis-promoting effect we observed. Indeed, splenectomy reduces the number of NK cells and increased metastasis formation in the liver; spleen was proposed as a source of factors that allow the traffic of NK cells into metastatic lesions^18^.

In contrast to our findings, splenectomy reduced lung metastases in other studies^9,13,21^. Although NK cells are well known to play a crucial role in controlling tumor metastasis, the sensitivity to NK cell cytotoxicity differs markedly depending on tumor cell types and their receptors they possess that control the function of NK cells^51–53^. In this study, the sensitivity of LuM1 cells to NK cells may have influenced the exacerbation of lung metastases due to the decrease in NK cells by splenectomy. In addition, metastases that arise from clusters of circulating tumor cells (CTCs) are much less sensitive to NK-mediated clearance than those that arise from single CTCs^54^. Moreover, NK cells effectively limit the pulmonary seeding of B16 melanoma cells, whereas T cells play a primary role in restricting the growth of metastatic foci in the lung^55^. Therefore, the apparently inconsistent effects of splenectomy on lung metastases may reflect the different sensitivities of various tumor cell types to NK cell-mediated lysis. The density of CD3^+^CD8^+^ T cells in metastatic lung tumors was increased in splenectomized mice, showing the same trend as that in primary tumors, and likely was due to the reduced number of M-MDSCs. Therefore, had we followed the splenectomized mice longer, we might have observed decreased growth of their metastatic lesions due to the cytotoxic effects of CD8^+^ TILs.

The mechanisms leading to the reduction in the number of circulating NK cells after splenectomy are unclear yet. According to a recent report, spleen-resident immature NK cells undergo extramedullary maturation in a T-bet–dependent manner, and the spleen is a key source of NK cells that can circulate peripherally and then migrate into the tumor tissue^56^. Importantly, patrolling monocytes promote NK cell recruitment and activation, which leads to the elimination of tumor cells in the lung^36^. In our current study, the proportion of patrolling monocytes correlated positively with that of NK cells and inversely with the number of lung metastases. The number of patrolling monocytes tended to be reduced in splenectomized mice, although the difference did not reach statistical significance. Given that the spleen is considered to be one of the main sites for the differentiation of patrolling monocytes^35^, decreased NK activity may be related to a reduced number of patrolling monocytes.

In the present study, the effect of the timing of splenectomy on tumor development could not be investigated. Previous studies have reported that the timing of splenectomy influences tumor progression^9,20,23,24^. Soda et al. investigated the effect of the timing of splenectomy on the survival in a murine colon cancer model by performing splenectomy either before or after tumor inoculation. In their study, splenectomy significantly prolonged the survival when it was performed prior to tumor inoculation, but not when performed after inoculation^12^. We performed splenectomy 3 weeks before tumor inoculation to minimize the effect of surgical stress on tumor development, however, the results may have been different if surgery had been performed after inoculation. Moreover, the method of tumor inoculation would also affect the tumor progression. Most of these previous studies used metastatic mouse models established by intravenous or intrasplenic injection of cancer cells^8,19,21^. In the present study, we subcutaneously inoculated mice with LuM1 cells to create a spontaneous lung metastasis model, allowing the effect of splenectomy on the primary tumor and lung metastases to be evaluated together. Given the differences in the microenvironment between the subcutaneous and the orthotopic sites, as well as metastatic sites, orthotopic inoculation (i.e., colon) or intravenous injection of LuM1 cells may have led to different tumor development. Our result shows that further research is needed to understand the potential role of splenectomy in therapy.

In summary, we found that the growth of LuM1 subcutaneous primary tumors was suppressed slightly, with reduced numbers of systemic M-MDSCs and M2-type TAMs and increased numbers of TILs, after splenectomy in BALB/C mice. These findings suggest that splenectomy enhances T cell-mediated immunity by inhibiting the expansion and systemic supply of M-MDSCs. In contrast, splenectomy enhanced lung metastasis, with decreased numbers of NK cells in peripheral blood and lung tissue, indicating that splenectomy suppresses NK cell-mediated immunity. The overall influence of splenectomy on a tumor-bearing host depends on the balance between these two different immune responses. A precise understanding of the susceptibility of each tumor type to NK- or T cell-mediated immunity will enable the development of next-generation cancer immunotherapy strategies.

## Materials and methods

### Reagents and antibodies

FITC-conjugated anti-CD49b (DX5) and anti-Ly6c (HK1.4); PE-conjugated anti-CD8a (53–6.7) and anti-CX3CR1 (SA011F11); APC-conjugated anti-CD11b (M1/70) and anti- CD335 (29A1.4); BV421-conjugated anti-CD4 (GK1.5) and anti-Gr-1 (RB6-8C5); and AlexaFluor 700-conjugated anti-CD3 (17A.2) antibodies were purchased from BioLegend (San Diego, CA, USA). FVS780 and BUV395-conjugated anti-CD45 (30-F11) and anti-CD192 (CCR2) (475301) antibodies were purchased from BD Biosciences (Franklin Lakes, NJ, USA). FcR blocking reagent was purchased from Miltenyi Biotec (Bergisch Gladbach, Germany). For immunohistochemistry, monoclonal antibodies (mAbs) to CD3e (SP7, rabbit IgG), CD8a (4SM15, rat IgG2a), and Ly6G/Ly6C (RB6-8C5, rat IgG2b) were purchased from Invitrogen (Santa Clara, CA, USA); to CD4 (EPR19514, rabbit IgG), CD163 (EPR19518, rabbit IgG), NCR1 (EPR23097-35, rabbit IgG), and granzyme B (EPR22645-206, rabbit IgG) from Abcam (Cambridge, MA, USA); and to F4/80 (BM8, rat IgG2a) from BioLegend. Signal Enhancer HIKARI for Immunostain Solution B (used as an antibody dilution buffer) and Blocking One Histo were obtained from Nacalai Tesque (Kyoto, Japan).

### Cell culture

LuM1, a highly lung metastatic subclone of the murine colon adenocarcinoma 26 line, was a kind gift from Dr. K. Oguri (Aichi Cancer Center, Nagoya, Japan)^33^ and was maintained in RPMI supplemented with 10% fetal bovine serum, 100U/mL penicillin, and 100 µg/mL streptolysin (Sigma- Aldrich, St. Louis, MO, USA). YTN16, a murine gastric cancer cell, established in p53 heterozygous knockout C57BL/6 mice by oral intake of *N*-Methyl-*N*-nitrosourea (MNU) was a generous gift from Dr S.Nomura (Tokyo University, Japan)^57^. YTN16P, a highly metastatic subline of YTN16 obtained through in vivo selection methods^58^, was cultured in DMEM supplemented with 10% et al bovine serum, 50 U/mL penicillin, and 50 µg/mL streptomycin. Once they had achieved > 80% confluence, cells were detached through treatment with TrypLE Express (Thermo Fisher Scientific, Waltham, MA, USA). Cultured cells were tested every 3 months by using the Mycoplasma Detection Kit (R&D Systems, Minneapolis, MN, USA) and used for experiments after three passages.

### Animal model

The experimental protocol is shown in Fig. 1A. Female BALB/c and C57BL/6N mice (age, 5 weeks) were purchased from CLEA Japan (Tokyo, Japan), housed under specific pathogen-free conditions, and underwent splenectomy (or sham operation) under anesthesia with isoflurane (Fujifilm Wako Chemical, Osaka, Japan) at 6 weeks of age as previously described^59^. Mice in the splenectomy group received an approximately 1-cm incision in the left flank, gentle mobilization of the spleen, ligature of splenic vessels, and removal of the spleen. In mice in the sham control group, the abdominal wall was opened, and the spleen was mobilized and manipulated gently for 3 min. Laparotomy wounds were closed in a continuous two-layer pattern. At 3 weeks after surgery, 1 × 10^6^ LuM1 or YTN16P cells in 100 µL HBSS were injected subcutaneously in the right flank of each mouse. Twice weekly thereafter, we weighed the mice, used calipers to measure the diameter of subcutaneous (primary) tumors, and calculated tumor volume (mm^3^) as [tumor length (mm) × tumor width (mm)^2^]/2.

At 4 (LuM1) or 8 (YTN16P) weeks after tumor inoculation, mice were euthanized through deep anesthesia by isoflurane. Immediately after euthanasia, blood samples (800 µL) were collected from the inferior vena cava, and 2 investigators blinded to treatment group independently weighed the primary tumor and counted the number of macroscopic metastatic lung nodules; the average of the two values was adopted for each parameter.

As syngeneic peritoneal metastasis model, YTN16P (1 × 10^6^ cells) was intraperitoneally injected into C57BL/6N mice at 3 weeks after surgery. At 2 weeks after tumor inoculation, mice were euthanized, and peritoneal metastases were assessed based on macroscopic nodules on the mesentery. All procedures were approved by the Animal Care Committee of Jichi Medical University (approval no. 21057-01) and performed in accordance with the ARRIVE guidelines and the Japanese Guidelines for Animal Research.

### Flow cytometry

For flow cytometry analysis, whole blood was harvested, red blood cells were eliminated with lysis buffer (NH_4_Cl, KHCO_3_, EDTA 4Na), and single-cell suspensions were prepared. Suspensions were adjusted to 1 × 10^6^ cells per 100 µL in PBS containing 0.02% EDTA, and incubated for 15 min at room temperature to label dead cells using FVS780. Cells were washed with PBS, incubated with 5 µL FcR blocking reagent for 10 min at 4 °C, and immunostained with relevant mAbs according to the manufacturer’s instructions. Cells were washed with PBS and then underwent flow cytometry (LSRFortessa X-20, Becton Dickinson, San Jose, CA, USA) for analysis of antigen expression by using Flow Jo software (Becton Dickinson). The absolute cell counts in 1 ml of blood were calculated by multiplying the specific subset frequency by the live cell counts obtained from microscopic observation using trypan blue staining.

### Sequential immunohistochemistry of subcutaneous primary tumors and lung samples

Mice were euthanized at day 28 after inoculation with tumor cells; subcutaneous primary tumors and lungs were excised and fixed with 4% formalin. Lungs also underwent internal fixation. Paraffin-embedded 4-µm representative central sections of samples were prepared for immunohistochemical evaluation and staining with hematoxylin and eosin.

Sequential immunohistochemistry was performed as previously described^60^. Briefly, after deparaffinization in xylene and rehydration through a graded series of ethanol immersion baths, sections were washed with running water for 5 min. After rehydration, slides were stained with hematoxylin for 1 min and then coverslips were applied with VectaMount AQ Aqueous Mounting Medium (Vector Laboratories, Newark, CA, USA). Slide then underwent whole-tissue scanning (OlyVia Slideview VS200, Olympus, Tokyo, Japan), after which coverslips were removed by immersion in Tris-buffered saline with 0.1% Tween 20 (TBST).

Endogenous peroxidases were blocked by incubating slides in 0.3% H_2_O_2_ for 30 min at room temperature. For antigen retrieval, the sections were processed by microwave method in Instant Buffer Phosphate Buffer Solution (As One, Osaka, Japan) for 10 min. Slides were washed in TBST and then incubated at room temperature in blocking agent (Blocking One Histo, Nacalai Tesque) to prevent nonspecific binding. The sections were then incubated for 30 min at room temperature with primary antibodies for CD3 (1:100 dilution), CD4 (1:1000), CD8 (1:100), CD163 (1:1000), F4/80 (1:50), Ly6G/Ly6C (1:50), NCR1 (1:500), and granzyme B (1:3000) thoroughly washed with TBST, and incubated for 30 min at room temperature with Histofine Simple Stain MAX PO for either rabbit or mouse (Nichirei, Tokyo, Japan), and primary antibody binding was visualized by using ImmPACT AMEC Red Substrate Lit (Vector Laboratories). The slides were coverslipped and scanned again.

After scanning, the coverslips were removed by using TBST and the slides were treated with a gradient of 70%, 80%, and 90% ethanol for every 2 min for destaining. Slides were incubated until no visible AEC reaction product remained. After destaining, antibodies were eluted by incubating sections in Instant Buffer Phosphate Buffer Solution (As One) for 10 min using the microwave method. The tissues were then revitalized, starting again with the blocking step, as described earlier. Complete stripping of antibodies and signals throughout all cycles was confirmed.

### Image processing and analysis

Image processing and analysis were performed as described previously^60^. In brief, iteratively digitized images were co-registered so that cell features overlapped at a single-pixel level by using the CellProfiler Version 4.2.1 pipeline Alignment_Batch.cppipe available under General Public License version 2.0. Visualization was performed via conversion of co-registered images to pseudo-colored single-marker images and merged by using ImageJ Fiji Version 2.9.0 (NIH, Bethesda, MD, USA). After processing, T cells, helper T cells, and cytotoxic T cells were identified as CD3^+^, CD3^+^CD4^+^ and CD3^+^CD8^+^ cells, respectively. Granulocytic MDSCs (G-MDSCs), NK cells, macrophages, and M2 macrophages were defined as Ly6G/Ly6C^+^, NCR1^+^, F4/80^+^, F4/80^+^CD163^+^ cells, respectively.

For evaluating the densities of these immune cells, we used a region of interest (ROI) of 1.0 × 1.0 mm for primary tumors and lung tissue; for lung metastatic nodules, we used an ROI of 0.3 × 0.3 mm for metastatic lesions larger than 160,000 µm^2^ (totals: 85 lesions in splenectomized mice, 26 in the sham group). Two investigators blinded to treatment group independently used ImageJ Fiji to count these cells in 3 random microscopic fields; they then calculated the density of each cell type (number/mm^2^).

### Statistical analysis

Statistical analysis was performed by using Prism 8 (Graph Pad Softeare, San Diego, CA, USA). Data are shown as median values or mean ± 1 standard deviation (SD) and were compared by using the Mann–Whitney *U* test. Correlation was evaluated through Pearson simple linear regression analysis. In all analyses, *P* < 0.05 was considered statistically significant.

### Animal study

The protocol of animal study was approved by Institution Review Board of the Animal Care Committee of Jichi Medical University (Approval No. 21057-01).

### Supplementary Information

Supplementary Information.

## Data Availability

All data generated or analysed during this study are included in this published article and its supplementary information files.
