# Supplementary material for: Splenectomy has opposite effects on the growth of primary compared with metastatic tumors in a murine colon cancer model
Source: Sci Rep. 2024 Feb 24;14:4496. doi: 10.1038/s41598-024-54768-5 (PMC10894273; doi:10.1038/s41598-024-54768-5)
Supplement: Supplementary file 1 — Supplementary Information. [file 41598_2024_54768_MOESM1_ESM.docx]

**
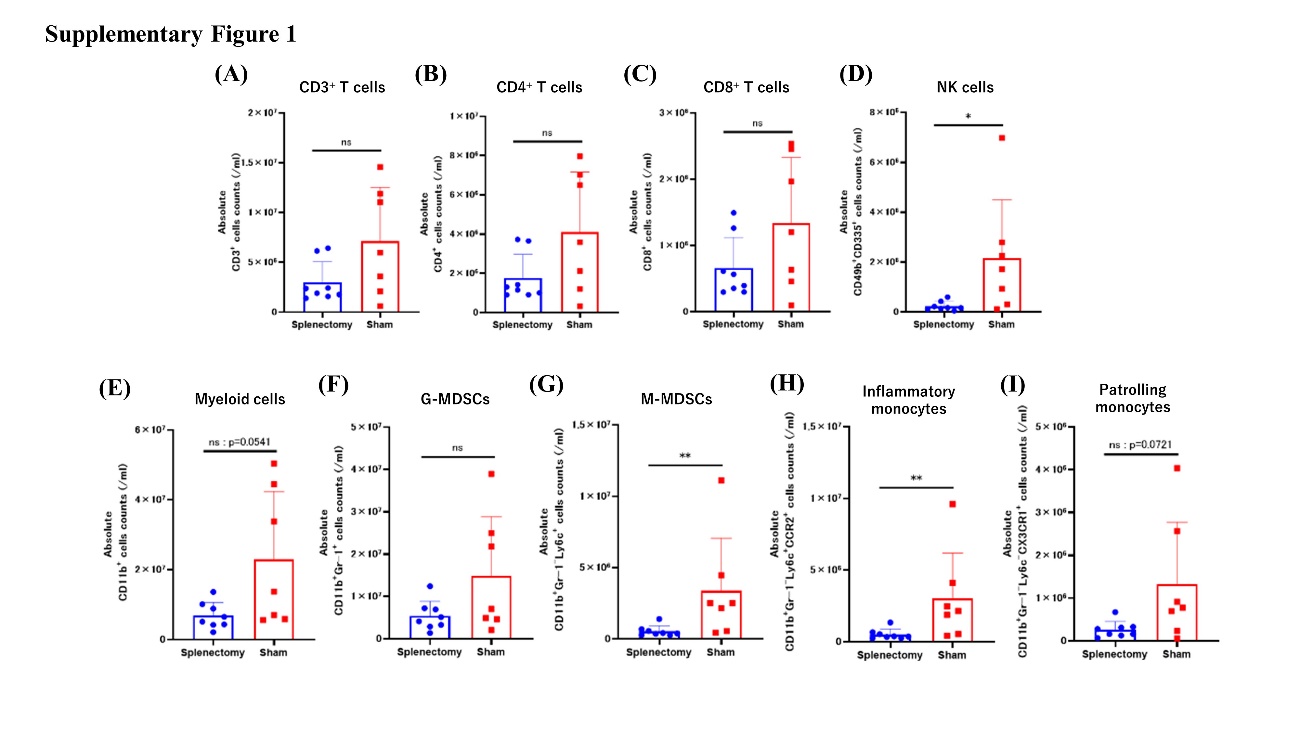
**

**Supplementary Figure 1. Absolute circulating lymphocyte and myeloid cell counts in blood.**

The frequencies of each cell subsets in live cell population were determined by using flow cytometry. The absolute cell counts in 1 ml of blood were calculated by multiplying the specific subset frequency by the live cell counts obtained from microscopic observation using trypan blue staining. Comparison of the absolute counts of (**A**) CD3^+^ total T cells, (**B**) CD3^+^CD4^+^ T cells, (**C**) CD3^+^CD8^+^ T cells, (**D**) CD49b^+^CD335^+^ NK cells, (**E**) CD11b^+^ myeloid cells, (**F**) CD11b^+^Gr-1^+^ granulocytic MDSCs, (**G**) CD11b^+^Gr-1^–^Ly6C^+^ monocytic MDSCs, (**H**) CD11b^+^Gr-1^–^Ly6C^+^CCR2^+^ inflammatory monocytes, and (**I**) CD11b^+^Gr-1^–^Ly6C^–^CX3CR1^+^ patrolling monocytes between splenectomized and control mice. * *P* < 0.05, ** *P* < 0.01 (Mann–Whitney *U* test).

**
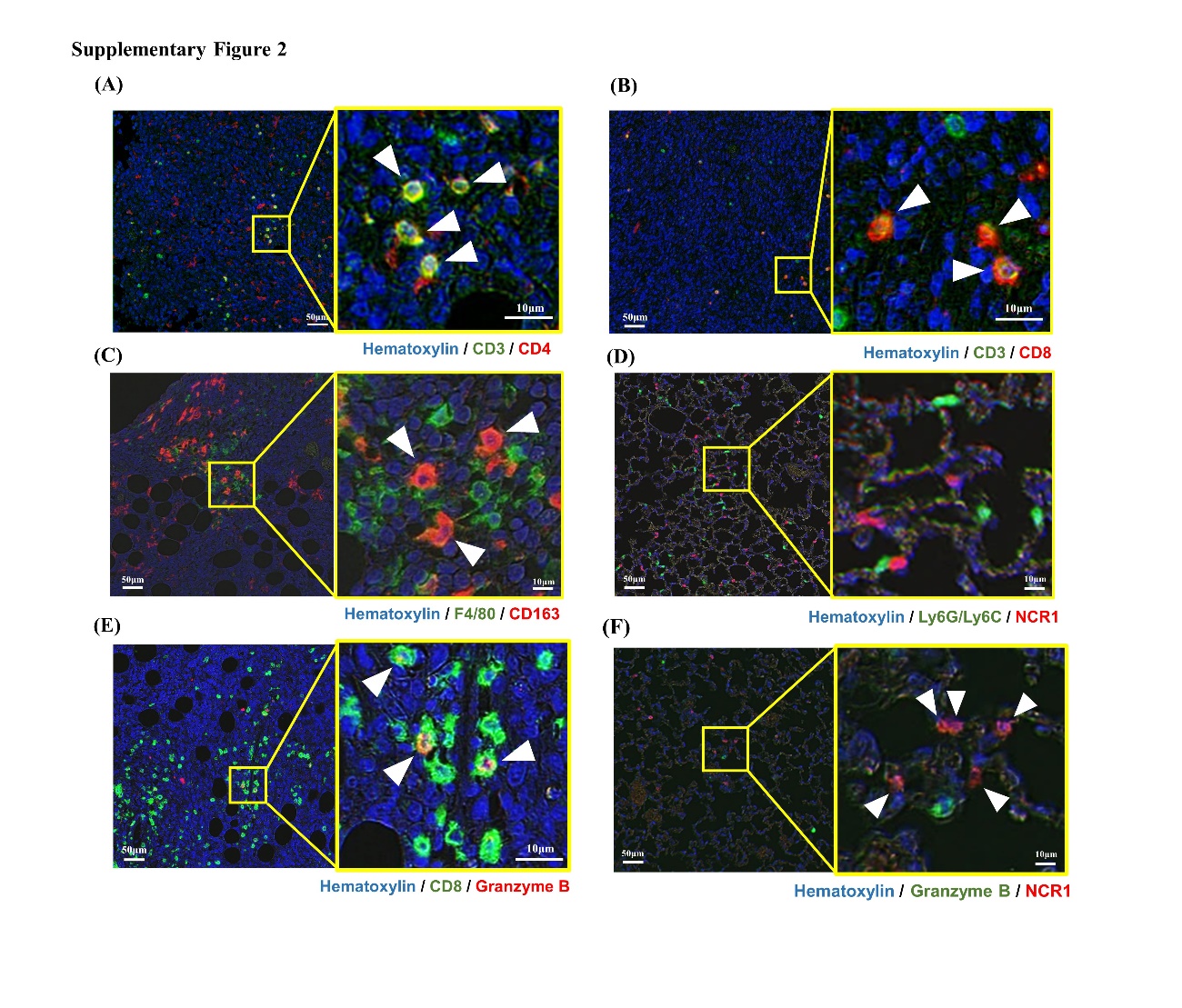
**

**Supplementary Figure 2. Representative images of multiplex immunostaining of T cells, macrophages, G-MDSCs, and NK cells in primary tumors or lung.**

CD4^+^ and CD8^+^ tumor-infiltrating T cells (TILs) were defined as (**A**) CD3(green)^+^CD4(red)^+^ and (**B**) CD3(green)^+^CD8(red)^+^ double-positive cells (arrow). (**C**) Tumor-associated macrophages (TAMs) were detected by F4/80(green)^+^ cells, and M2-type TAMs were defined as F4/80(green)^+^CD163(red)^+^ double-positive cells (arrow). (**D**) G-MDSCs and NK cells were identified as Ly6G/Ly6C(green)^+^ and NCR1(red)^+^ cells, respectively. (**E**) Granzyme B^+^ CD8^+^ TILs were identified as CD8(green)^+^Granzyme B(red)^+^ double-positive cells (arrow). (**F**) Granzyme B^+^ NCR1^+^ TILs were identified as granzyme B(green)^+^NCR1(red)^+^ double-positive cells (arrow). The majority of NCR1^+^ NK cells expressed granzyme B in metastatic lung.

**
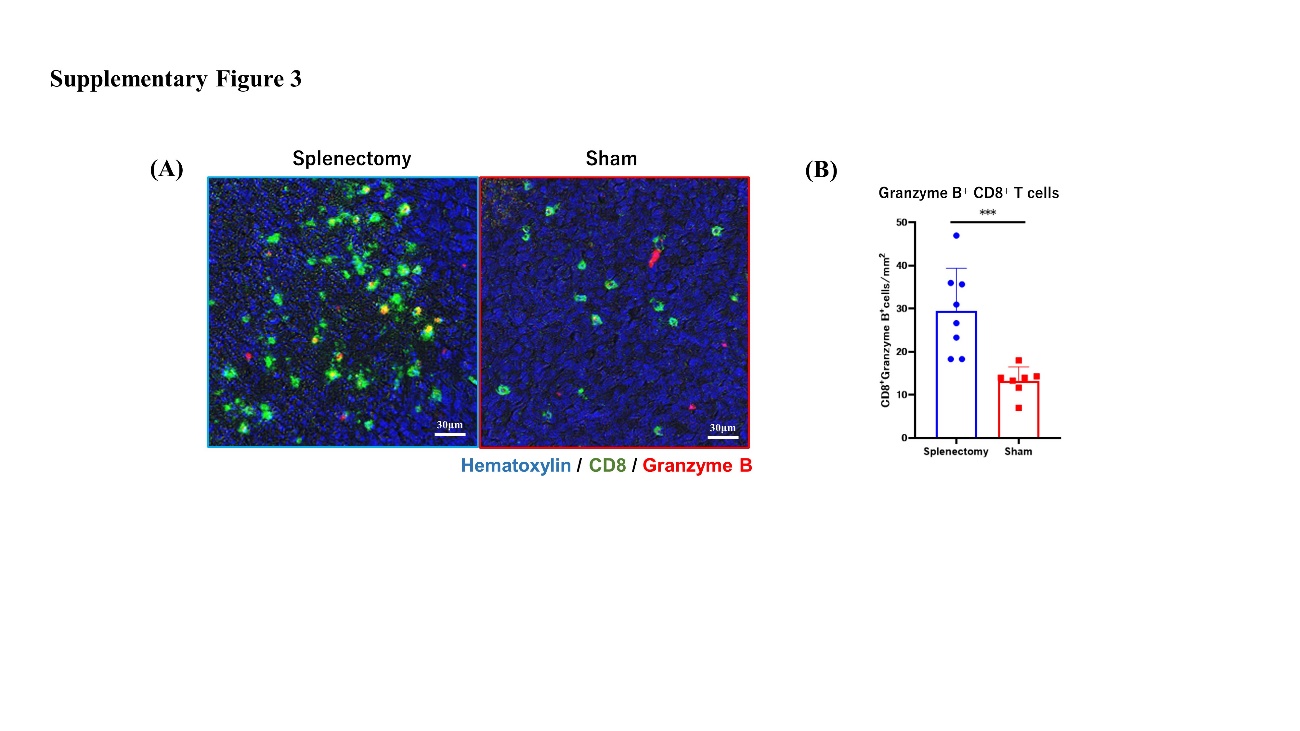
**

**Supplementary Figure 3.** **Multiplex immunostaining of granzyme B^+^ CD8^+^ TILs in primary tumors.**

(**A**) CD8(green)^+^ granzyme B(red)^+^ TILs in 3 randomly selected fields (1.0×1.0mm) of primary tumors were counted. (**B**) Comparison of the density of the type of TILs between splenectomized and control mice. *** *P* < 0.001 (Mann–Whitney *U* test).

**
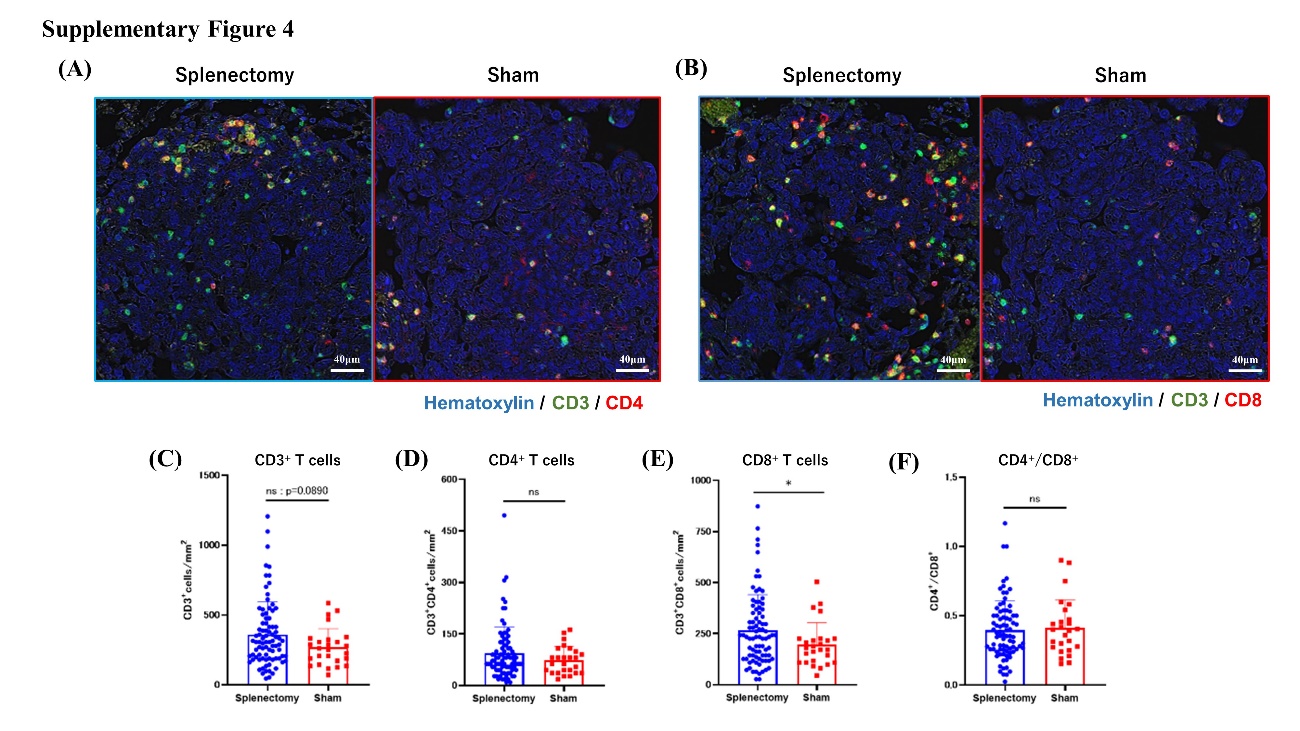
**

**Supplementary Figure 4.** **Multiplex immunostaining of tumor-infiltrating lymphocytes (TILs) in metastatic lung nodules.**

CD4^+^ and CD8^+^ TILs were defined as (**A**) CD3(green)^+^CD4(red)^+^ and (**B**) CD3(green)^+^CD8(red)^+^ double-positive cells. The numbers of these cells in 0.3×0.3 mm in metastatic lung lesions larger than 160,000 µm^2^ (total lesions: 85 in splenectomy group, 26 in controls) were counted. (**C–F**) The densities of CD3^+^, CD4^+^, and CD8^+^ TILs and the CD4^+^/CD8^+^ ratio were compared between splenectomized and control mice. * *P* < 0.05, ** *P* < 0.01, *** *P* < 0.001 (Mann–Whitney *U* test).


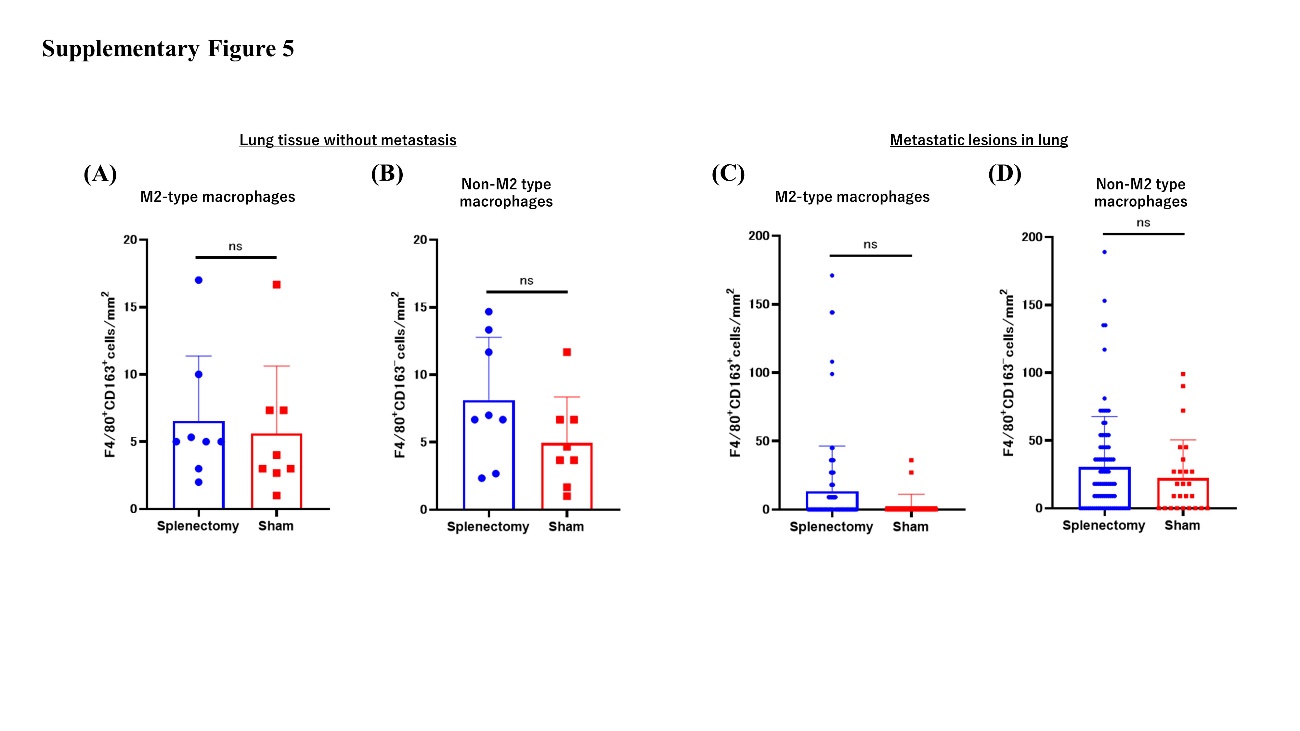


**Supplementary Figure 5.** **Multiplex immunostaining of tumor-associated macrophages (TAM) in lung.**

F4/80^+^CD163^+^ double-positive M2-type TAMs and F4/80^+^ TAMs in 3 randomly selected fields (**A,** **B**) 1.0×1.0mm in lung tissue without metastasis or (**C, D**) 0.3×0.3mm in metastatic lesions larger than 160,000 µm^2^ (total lesions: 85 in splenectomy group, 26 in control group) were counted. Comparison of the density of each TAM population was compared between splenectomized and control mice (Mann-Whitney *U* test).


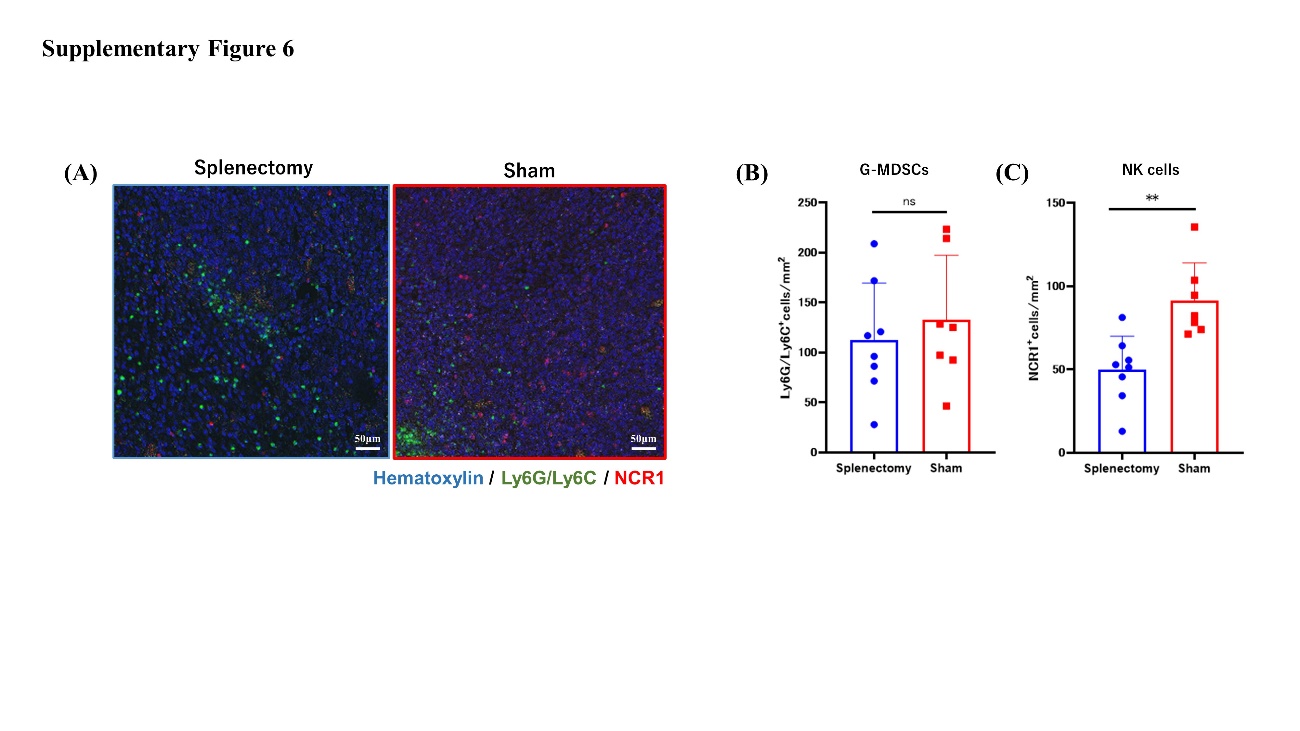


**Supplementary Figure 6.** **Multiplex immunostaining of granulocytic MDSCs (G-MDSCs) and NK cells in primary tumors.**

(**A**) Ly6G/Ly6C(green)^+^ G-MDSC and NCR1(red)^+^ NK cells in 3 randomly selected fields (1.0×1.0mm) of primary tumors were counted. Comparison of the density of (**B**) G-MDSCs and (**C**) NK cells between splenectomized and control mice. ** *P* < 0.01 (Mann–Whitney *U* test).


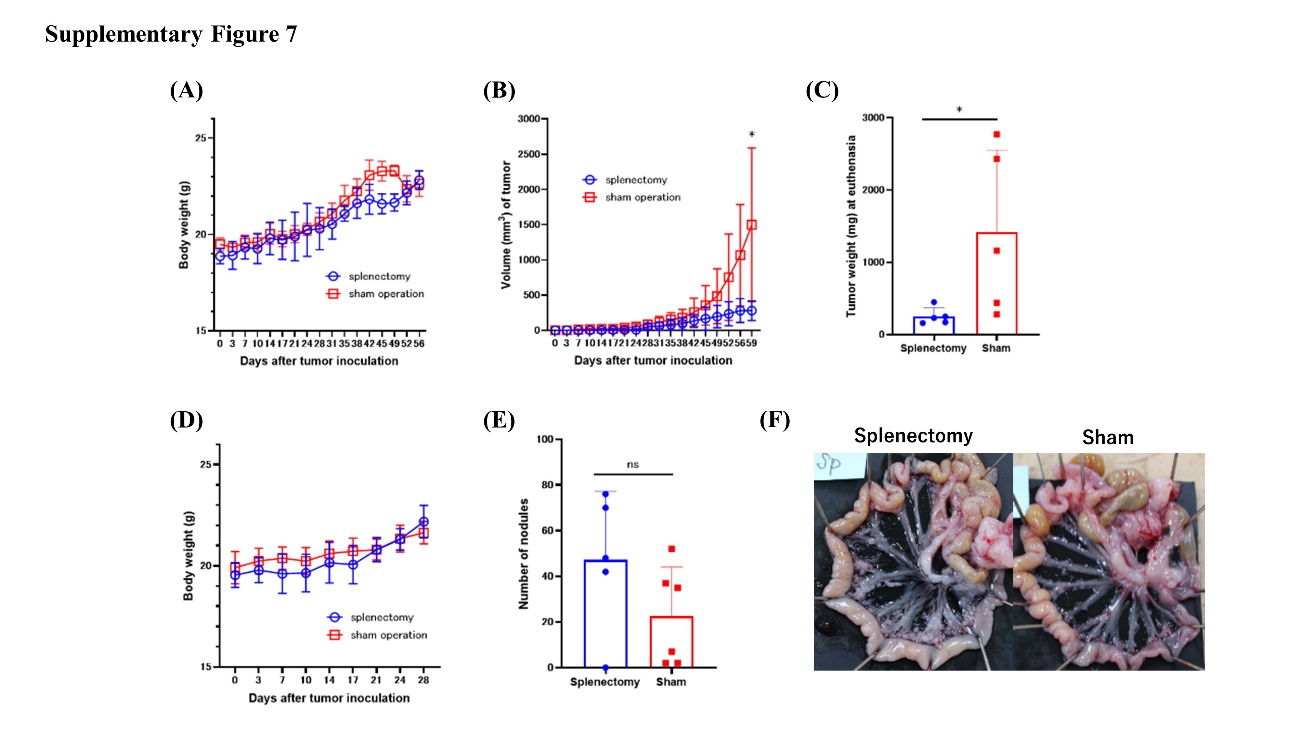


**Supplementary Figure 7. Subcutaneous tumor growth and the formation of peritoneal metastases in a gastric cancer model.**

C57BL/6N mice underwent splenectomy or a sham operation prior to subcutaneous injection of YTN16P gastric adenocarcinoma cells (1 × 10^6^ per mouse) in the right flank. (**A**) Body weight and (**B**) tumor volume were measured twice each week after tumor inoculation. On day 59, mice were euthanized, and (**C**) primary tumors were weighed (n = 5 in each group). No lung metastases were found in both groups. As syngeneic peritoneal metastasis model, YTN16P (1 × 10^6^ per mouse) was intraperitoneally injected into C57BL/6N mice. (**D**) Body weight was also measured twice each week. (**E, F**) On day 28, peritoneal metastases were assessed based on macroscopic nodules on the mesentery (n = 5 in each group). * *P* < 0.05 (Mann–Whitney *U* test).
